# Taxonomy of the genus *Homalota* Mannerheim in Korea (Coleoptera, Staphylinidae, Aleocharinae)

**DOI:** 10.3897/zookeys.447.7728

**Published:** 2014-10-16

**Authors:** Yoon-Ho Kim, Kee-Jeong Ahn

**Affiliations:** 1 Department of Biology, Chungnam National University, Daejeon 305-764, Republic of Korea

**Keywords:** Staphylinidae, Aleocharinae, *Homalota*, key to species, Korea, new combination, taxonomy

## Abstract

A taxonomic study of the genus *Homalota* Mannerheim in Korea is presented. Five species are recognized, one of which, *Homalota serrata* (Assing), **comb. n.** is transferred from *Anomognathus* Solier. *Homalota fraterna* (Sharp), *Homalota mikado* Likovský, *Homalota sauteri* Bernhauer, and *Homalota serrata* are newly added to the Korean fauna. A key, descriptions, and illustrations of the diagnostic characters are provided.

## Introduction

The genus *Homalota* Mannerheim in the tribe Homalotini Heer contains 72 species worldwide, with six species recorded from the Palearctic region (Smetana 2004, Newton and Thayer 2005). In East Asia, three species have been recorded from Japan and Taiwan, and two species from China. In the Korean Peninsula, Paśnik (2001) reported *Homalota plana* (Gyllenhal) from North Korea. Members of *Homalota* are usually found under bark, but are often collected from decaying fruits or using flight intercept trap (FITs).

During an ongoing study of the Korean Homalotini, we recognized five *Homalota* species. We concluded that *Anomognathus serratus* Assing should be transferred to the genus *Homalota*. *Homalota fraterna* (Sharp), *Homalota mikado* Likovský, *Homalota sauteri* Bernhauer, and *Homalota serrata* are new to the Korean fauna. We provide a key, descriptions, and illustrations of the diagnostic characters for these five species.

## Methods

The specimens were examined by scanning electron microscopy (SEM; S-4800, Hitachi High-Technologies, Tokyo, Japan). Specimens were dissected in water and mounted on sticky carbon tape. Then, the specimens dried at 60 °C on a slide warmer for 24 h, sputter-coated with Pt/Pd nanoparticles using a sputter coater (208 HR, Cressington Scientific Instruments, Watford, Hertfordshire, UK), and examined with SEM. The terminology used here follows Sawada (1972), but we followed Ashe (1984) in some cases. Ashe (1984) modified Sawada's character system, especially the mouthparts, to reduce some confusion. North Korean species were borrowed from the Institute of Systematics and Evolution of Animals (ISEA), Kracôw, Poland. All other examined specimens are deposited in the Chungnam National University Insect Collection (CNUIC), Daejeon, Korea.

## Results

### Genus Homalota

Taxon classificationAnimaliaColeopteraStaphylinidae

Mannerheim

Homalota Mannerheim, 1830: 73. Type species. *Aleochara plana* Gyllenhal, 1810.

#### Diagnosis.

Body strongly to moderately dorsoventrally flattened, parallel-sided. Head almost as wide as pronotum or slightly narrower than pronotum. Eye moderate in size, almost as long as tempora. Infraorbital carina well developed, complete. Labium with two palpomeres, ligula as long as palpomere 1, its apical half bifid. Scutellum postero-medially round. Mesocoxae narrowly separated, isthmus present, less than about half of mesocoxae length. Male tergite VIII with modified processes in males (most species) or in both sexes (some species).

#### Key to the species of the genus *Homalota* Mannerheim in Korea

**Table:** 

1	Body dorsoventrally slightly flattened; mesocoxae moderately separated (Fig. 17)	***Homalota mikado***
–	Body dorsoventrally strongly flattened; mesocoxae very narrowly separated (Fig. 10)	**2**
2	Body yellowish brown; size smaller, length less than 2.1 mm	**3**
–	Body brown to dark brown; size larger, length more than 2.1 mm	**4**
3	Head narrower than pronotum; male tergite VII with 16 to 18 tubercles (Fig. 27)	***Homalota sauteri***
–	Head almost as wide as pronotum; male tergite VII without tubercles	***Homalota serrata***
4	Male tergite VII with a distinct tubercle (Fig. 21); male tergite VIII without lateral process (Fig. 22); tergite X with medial setal patch subpentagon, loss of setae postero-medially	***Homalota plana***
–	Male tergite VII without tubercle; male tergite VIII with short lateral processes (Fig. 11); tergite X with medial setal patch subquadrate, with transverse row of large spines posteriorly	***Homalota fraterna***

### Homalota fraterna

Taxon classificationAnimaliaColeopteraStaphylinidae

(Sharp, 1888)

Figs 1 , 6 – 14

Epipeda fraterna Sharp, 1888: 376.
Homalota fraterna : Fenyes 1914: 46; Fenyes 1918: 87; Smetana 2004: 448.

#### Specimens examined.

KOREA: Chungbuk Prov., Yeongdong-gun, Sangchon-myeon, Mulhan-ri, Mt. Minjujisan, 36°03'35.2"N, 127°52'31.3"E 518 m, 18 V 2011, JG Lee, TK Kim, decaying persimmon (16 exx., 3♂♂, 2♀♀ on slides); Jeonnam Prov., Hadong-gun, Hwahye-myeon, Ssanggyesa, 25 V 2000, K.-J. Ahn, *ex* under bark (3♀♀); Kangwon Prov., Hoengsunggun, Unduryeng, 9–10 ix 1998, K.-J. Ahn, *ex* under bark (1♂); Jeju Prov., Seongpanak, 28 IV 1985, KS Lee (3♂♂, 1♀, 1♂ on slide).

#### Description.

**Figures 1–5.**
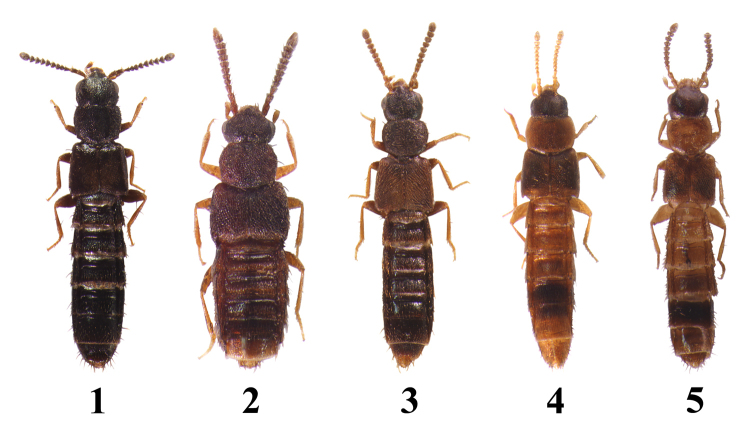
Habitus: **1** *Homalota fraterna*, 2.3 mm **2** *Homalota mikado*, 2.0 mm **3** *Homalota plana*, 2.9 mm **4** *Homalota sauteri*, 1.8 mm **5** *Homalota serrata*, 2.1 mm.

**Figures 6–11.**
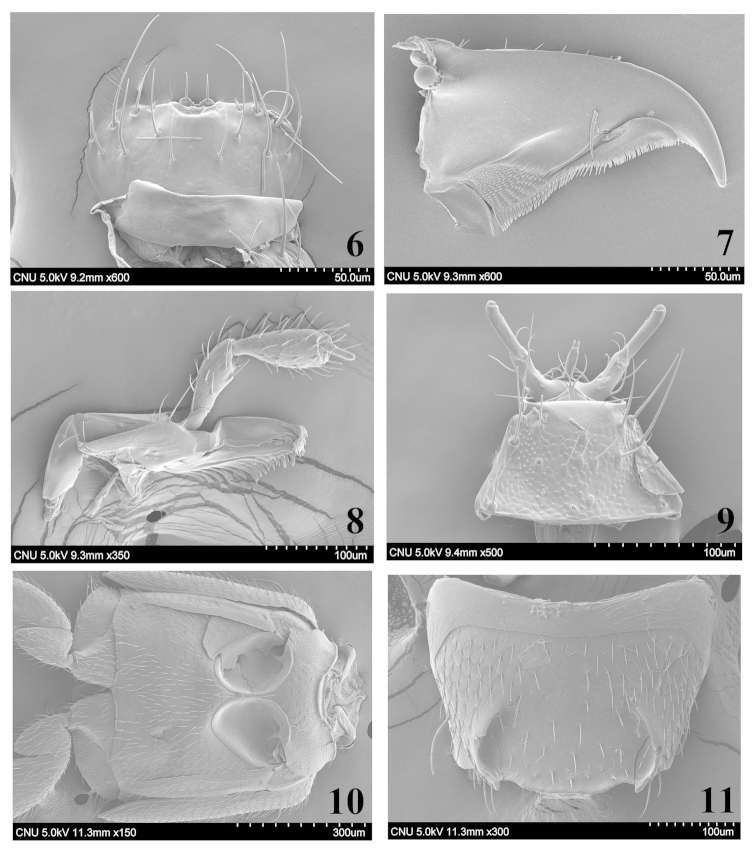
SEM photos, *Homalota fraterna*: **6** labrum, ventral aspect **7** right mandible, ventral aspect **8** maxilla, ventral aspect **9** labium, ventral aspect **10** meso- and metaventrites, ventral aspect **11** male tergite VIII, dorsal aspect.

**Figures 12–14.**
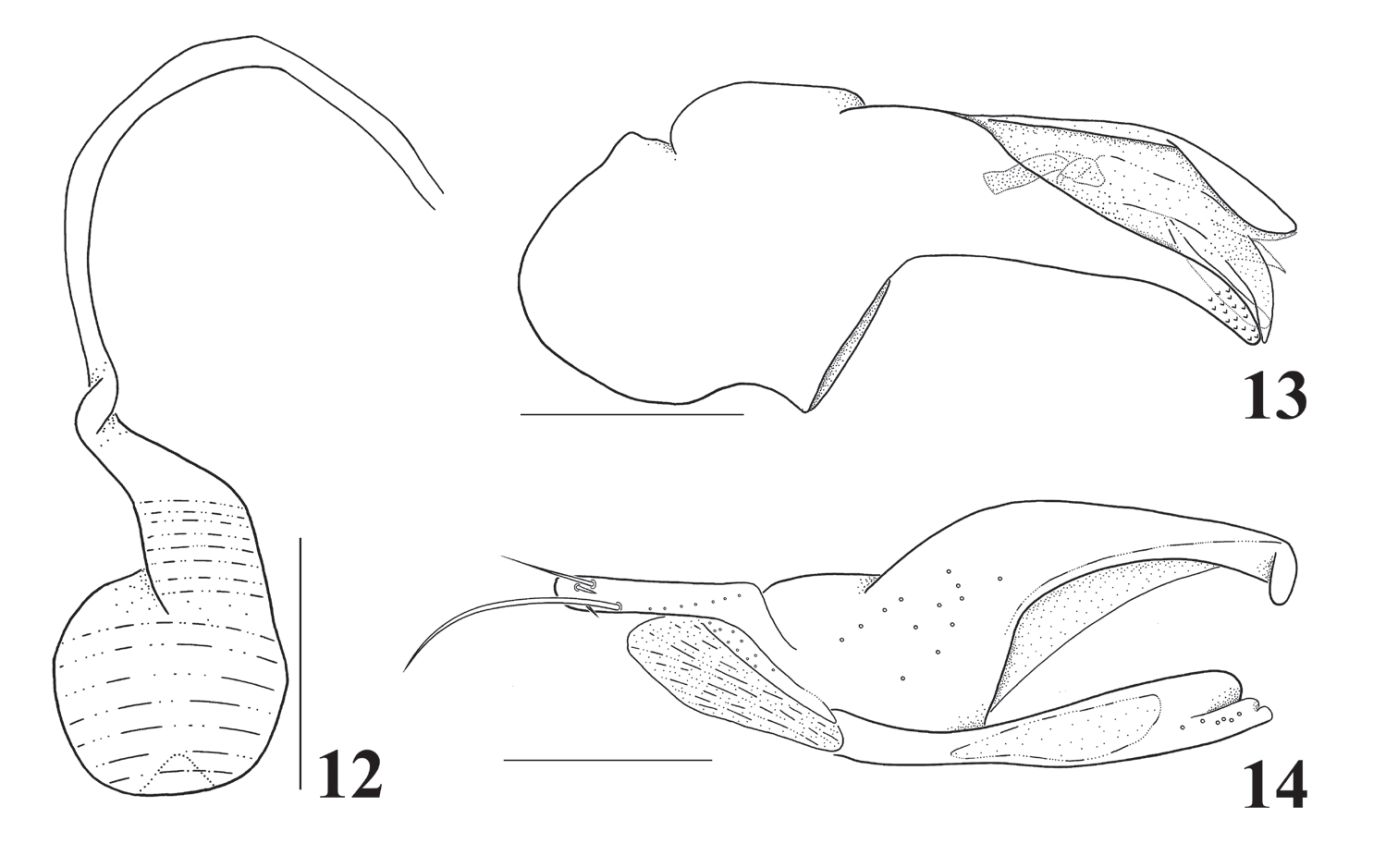
*Homalota fraterna*: **12** spermatheca **13** median lobe, lateral aspect **14** paramere, lateral aspect. Scale bars 0.1 mm.

Body length 2.3–2.6 mm (Fig. 1). Body dark brown, antennae and legs brown; dorsoventrally flattened, parallel-sided; surface subglossy, slightly pubescent. *Head.* Subquadrate, almost as wide as pronotum; eyes moderate in size, as long as tempora; infraorbital carina well developed, complete; antennomeres 4–10 transverse, 5–10 slightly incrassate toward apex. *Mouthparts.* Labrum (Fig. 6) transverse, 7 pairs of macrosetae present, sensilla of antero-medial sensory area shallow and narrowly emarginated, α-sensillum with a setose process, β and γ minute and conical, ε with a short setose process, distinctly shorter than α; two lateral sensilla present on lateral margins of epipharynx, without transverse row of sensory pores on basal region of epipharynx; right mandible (Fig. 7) with small median tooth, prostheca well developed; maxillary palpomere (Fig. 8) 2 and 3 dilated distally, 4 without small spines at apex; labium (Fig. 9) with ligula moderate in length and bifid in its apical half, almost as long as labial palpomere 1, labial palpus with two palpomeres, palpomere 1 almost as long as 2, two medial setae present on prementum, contiguous and one laterally behind the other, median pseudopore field of prementum narrow and with pseudopores, mentum not emarginated in anterior margin. *Thorax.* Pronotum slightly transverse, about 1.25 times wider than long, widest at apical third, surface pubescent, directed anteriorly in narrow median strip and directed antero-laterally to laterally in lateral area, with some distinct macrosetae, hypomeron broadly visible in lateral aspect; prosternum with a median knob; elytra slightly wider than pronotum, postero-laterally slightly sinuate; mesoventrite (Fig. 10) without longitudinal carina, mesoventral process narrow, apex point; metaventral process round at apex, distinctly shorter than mesoventral process; isthmus present; mesocoxae narrowly separated; tarsomere 1 as long 2, without empodial seta between tarsal claws. *Abdomen.* Tergites III–VI transversely impressed; tergite X with medial setal patch subquadrate, with transverse row of large spines posteriorly, 4 macrosetae on each side. *Genitalia.* Spermatheca (Fig. 12) simple and elongate at base; median lobe (Fig. 13) bulbous at base, apical process slender and short with some tubercles, distinctly shorter than basal bulb, flagellum well sclerotized and short; paramere (Fig. 14) with apical lobe of paramerite subcylindrical, with four setae, basal one largest, condylite subequal in length to apex of paramerite. *Secondary sexual characteristics.* Posterior margin of male tergite VIII (Fig. 11) with two short lateral processes, middle margin broadly round.

#### Distribution.

Korea (South), China (Hainan), Japan, Taiwan.

#### Remarks.

*Homalota fraterna* is similar to *Homalota plana*, but can be distinguished by the following features: antennomere 4 transverse, male tergite VII without tubercle, male tergite VIII with short lateral processes; tergite X with medial setal patch subquadrate, with transverse row of large spines posteriorly.

### Homalota mikado

Taxon classificationAnimaliaColeopteraStaphylinidae

Likovský, 1984

Figs 2 , 15 – 20

Homalota mikado Likovský, 1984: 6 [Replacement name]; Smetana 2004: 448.
Epipeda granigera Sharp, 1888: 375 [Homonym].
Homalota granigera : Fenyes 1914: 45.

#### Specimens examined.

KOREA: Gangwon Prov., Pyeongchang-gun, Pyeongchang-eup, Noron-ri, Mt. Sambangsan, 13 VII–15 VIII 2001, KJ Ahn, SJ Park, CW Shin, *ex* FIT (2 exx., 1♀ on slide); Mt. Bokjusan, Seo-myeon, Cheolwon-gun, N38°08'38.2"E127°28'26.7"138 m, 25 IX 2005, YB Cho (2 exx.); Jeonnam Prov., Jangseong-gun, Mt. Naejangsan, Baekyangsa Area, 25 VI 2000, HJ Kim, *ex* sifting (3 exx., 1♂ on slide); Chungbuk Prov., Mt. Sokrisan, Beobjusa Temple, Naesokri-myeon, Boeun-gun, *ex* FIT, 1–31 V 2007, YB Cho, 36°32'21.5"N, 127°50'10.4"E (1 ex.).

#### Description.

**Figures 15–20.**
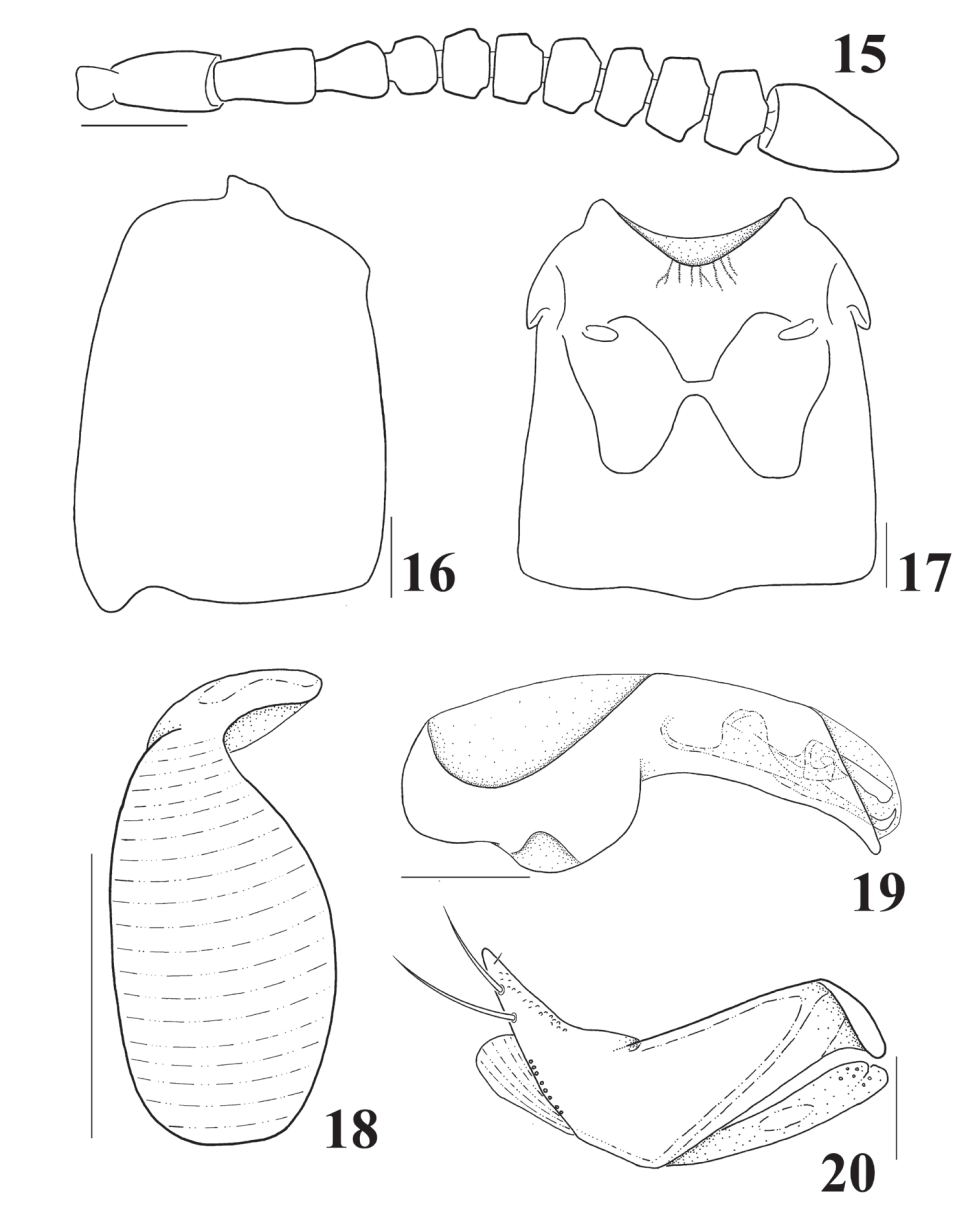
*Homalota mikado*: **15** antenna **16** elytron, dorsal aspect **17** meso- and metaventrites, ventral aspect **18** spermatheca **19** median lobe, lateral aspect **20** paramere, lateral aspect. Scale bars 0.1 mm.

Body (Fig. 2) length about 1.8–2.1 mm. Body dorsoventrally slightly flattened, widest at posterior margin of elytra; surface punctuate, subglossy, slightly pubescent; light brown to brown, abdominal tergite VI dark brown. *Head.* Subquadrate, slightly narrower than pronotum; eyes moderate in size, as long as tempora; head narrowed from behind of eyes to apical half of tempora but rarely narrowed to occipital construction; antennomere (Fig. 15) 4 slightly transverse, 5–10 transverse, slightly incrassate toward. *Mouthparts.* Labrum transverse, 8 pairs of macrosetae present, sensilla of antero-medial sensory area distinct, shallowly and width moderately emarginated; α-sensillum with a setose process, β and γ minute and conical, ε with a minute setose process, distinctly shorter than α, two lateral sensilla present on lateral margins of epipharynx, transverse row of sensory pores absent on basal region of epipharynx; right mandible with small median tooth, prostheca well developed, divided into 3 distinct area; maxillary palpomeres 2–3 dilated distally, 4 without small spine at apex; labium with ligula slender and elongated, bifid at apical third, almost as long as labial palpomere 1, labial palpus with two palpomeres, 1 longer than 2, two medial setae contiguous on prementum, side by side, median pseudopore field of prementum narrow and with pseudopores, mentum strongly emarginated in anterior margin. *Thorax.* Pronotum transverse, about 1.3 times wider than long, widest at apical third, surface pubescent, without distinct macrosetae; hypomeron broadly visible in lateral aspect; prosternum with a median knob; elytra (Fig. 16) wider than pronotum, postero-laterally sinuate; wings fully developed; mesoventrite (Fig. 17) without longitudinal carina, mesoventral process truncate at apex; metaventral process round at apex, longer than metaventral process; isthmus present; mesocoxae moderately separated; tarsomere 1 of front leg as long as 2, 1 slightly longer than 2 in middle and hind legs, without empodial setae between tarsal claws. *Abdomen.* Tergites III–VI transversely impressed; tergite X with medial setal patch subquadrate, with 3 macrosetae on each side. *Genitalia.* Spermatheca (Fig. 18) simple and elongate at base, duct short; median lobe (Fig. 19) bulbous at base, apical process slender and elongate, longer than basal bulb, flagellum well sclerotized and short; paramere (Fig. 20) with apical lobe of paramerite with four setae, two setae longer than others, condylite subequal in length to apex of paramerite. *Secondary sexual characteristics.* Absent.

#### Distribution.

Korea (South), Japan, Taiwan.

#### Remarks.

The taxonomic position of *Homalota mikado* is unclear, considering the numerous characters that distinguish it from other *Homalota* species, including the type species *Homalota plana*: body not subparallel-sided; surface with numerous large punctures; labrum with 8 pairs of macrosetae; ligula slender and elongate, mentum deeply emarginated in anterior margin; mesocoxae moderately separated. However, we are not sure what other aleocharine genus it may belong to. Therefore, the position of *Homalota mikado* in *Homalota* is tentatively maintained here, pending further comprehensive research of this species.

### Homalota plana

Taxon classificationAnimaliaColeopteraStaphylinidae

(Gyllenhal, 1810)

Figs 3 , 21 – 25

Aleochara plana Gyllenhal, 1810: 402.
Homalota plana : Mannerheim 1830: 73; Fenyes 1918: 87; Smetana 2004: 448.
Homalota plana See Smetana (2004) for additional references.

#### Specimens examined.

KOREA: Cerjong, Hvanghe-pukto, IX 1971, leg. J. Pawlowski, *Homalota plana* (Gyllenhal), det. G. Paśnik, 2000, Col ISEZ K-ow from box-no: KOR 1, Species name in box: *Homalota plana* (2♂♂, 2♀♀) (ISEA); HUNGARY: Ócsa, Pest m, 30 X 1952, nyárfakéreg, alólrostálva, leg Kaszab Z., *Homalota plana* (Gyll.) Det.: Adàm, 1987 (17 exx., 1♂, 1♀ on slides).

#### Description.

**Figures 21–25.**
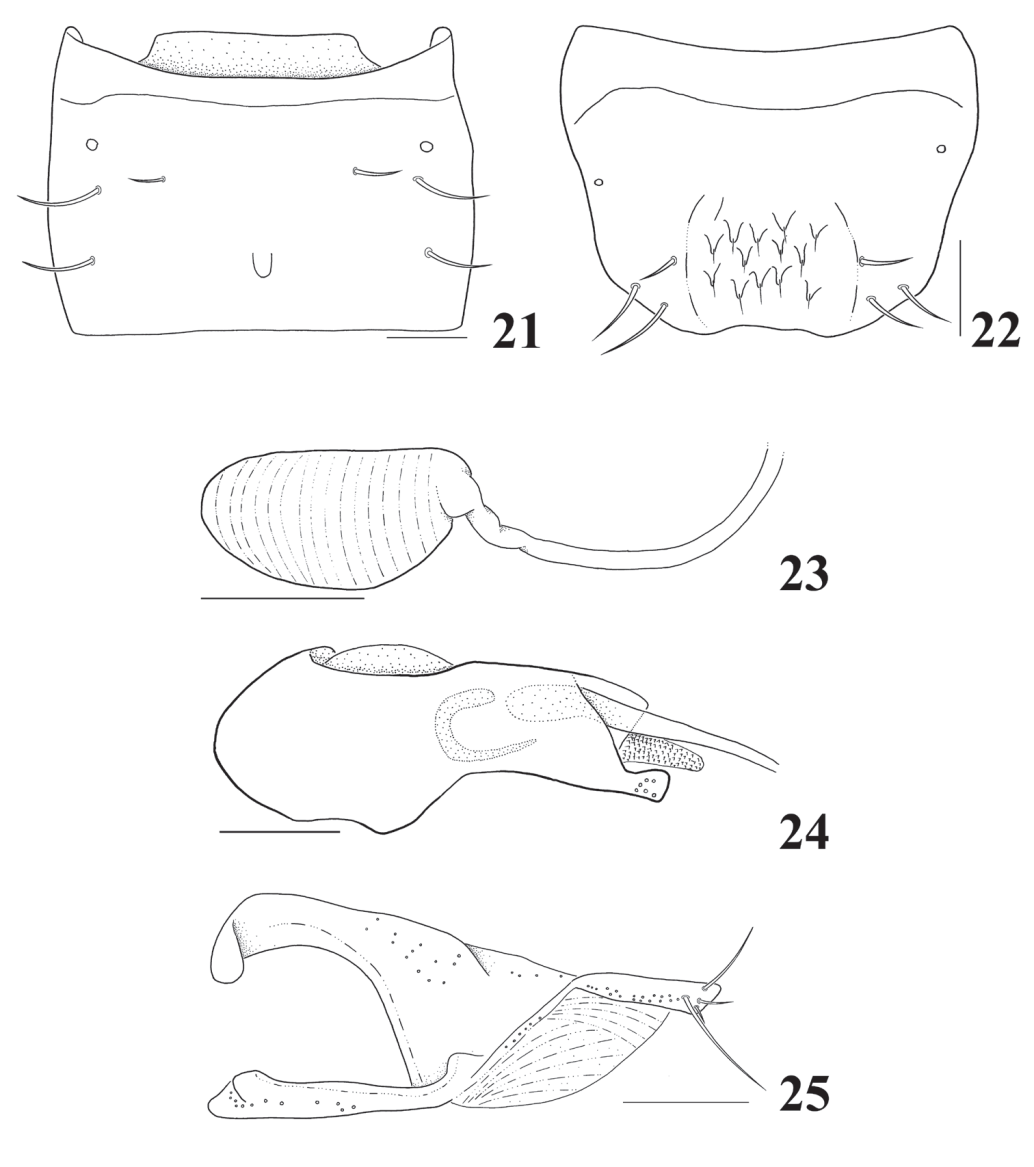
*Homalota plana*: **21** male tergite VII, dorsal aspect **22** male tergite VIII, dorsal aspect **23** spermatheca **24** median lobe, lateral aspect **25** paramere, lateral aspect. Scale bars 0.1 mm.

Body (Fig. 3) length about 2.4–3.3 mm. Body dorsoventrally flattened, subparallel-sided; subglossy, slightly pubescent; dark brown, antennae, elytra and legs brown. *Head.* Subquadrate, narrower than pronotum; eyes moderate in size, almost as long as tempora; antennomeres 4–10 slightly transverse. *Mouthparts.* Labrum transverse, 7 pairs of macrosetae present, sensilla of antero-medial sensory area distinct, depth and width moderately emarginated; α-sensillum with a setose process, β and γ minute and conical, ε with a short setose process, distinctly shorter than α, two lateral sensilla present on lateral margins of epipharynx, transverse row of sensory pores absent on basal region of epipharynx; right mandible with small median tooth, prostheca well developed, divided into 3 distinct area; maxillary palpomere 2 dilated distally, 3 distinctly dilated to apical third and then slightly convergent toward apex, 4 without small spine at apex; labium with ligula moderate and bifid at half, slightly shorter than labial palpomere 1, labial palpomere 1 slightly longer than 2, two medial setae contiguous on prementum, one laterally behind the other, median pseudopore field of prementum narrow and with pseudopores, mentum slightly emarginated in anterior margin. *Thorax.* Pronotum slightly transverse, about 1.2 times wider than long, widest at half, surface pubescent, directed postero-laterally; hypomeron visible in lateral aspect; prosternum with a median knob; elytra wider than pronotum, postero-laterally slightly sinuate; mesoventrite without longitudinal carina, mesoventral process narrow, apex narrowly round; metaventral process round at apex, distinctly shorter than mesoventral process; isthmus present; mesocoxae narrowly separated, tarsomere 1 as long as 2, with an empodial seta between tarsal claws. *Abdomen.* Tergites III–VI transversely impressed; tergite X with a medial setal patch subquadrate, with 4 macrosetae on each side. *Genitalia.* Spermatheca (Fig. 23) simple and elongate, tube slightly curved; median lobe (Fig. 24) elongate, bulbous at base, apical process slender and elongate, surface with some tubercles, flagellum well sclerotized and moderately long; paramere (Fig. 25) with apical lobe of paramerite subcylindrical, with four setae, two distinctly smaller than others, condylite subequal in length to apex of paramerite. *Secondary sexual characteristics.* Posterior margin of male tergite VII (Fig. 21) with a more or less distinct tubercle (occasionally missing); male tergite VIII (Fig. 22) impressed postero-medially, apex emarginated and without lateral process.

#### Distribution.

Korea (North). See Smetana (2004) for additional distribution.

### Homalota sauteri

Taxon classificationAnimaliaColeopteraStaphylinidae

Bernhauer, 1907

Figs 4 , 26 – 29

Homalota sauteri Bernhauer, 1907: 391; Fenyes 1918: 87; Smetana 2004: 448.

#### Specimens examined.

KOREA: Jeonnam Prov., Jangseon-gun, Jangseon-eup, Yutang-ri, N35°18‘54.8“E126°48‘34.0“90m, 22 V 2007, TK Kim, YH Kim, *ex* under bark (5 exx.); Chungnam Prov., Daejeon city, Sutongol, 18 IV 1998, KJ Ahn, HJ Kim, HJ Kim, KL Yu, *ex* sifting (2 exx.); Daejeon-si, Dong-gu, Secheon-dong, Mt. Sikjangsan, 36°19'34.7"N, 127°29'1.4"E 156 m, 11 IV 2010, IS Yoo, SG Lee, under bark (2 exx.); Gangwon Prov., Sokcho-city, Mt. Seolak, Hwa-amsa, 21 VI 2002, SJ Park, CW Shin, ex fungus on log (2 exx.).

#### Description.

**Figures 26–29.**
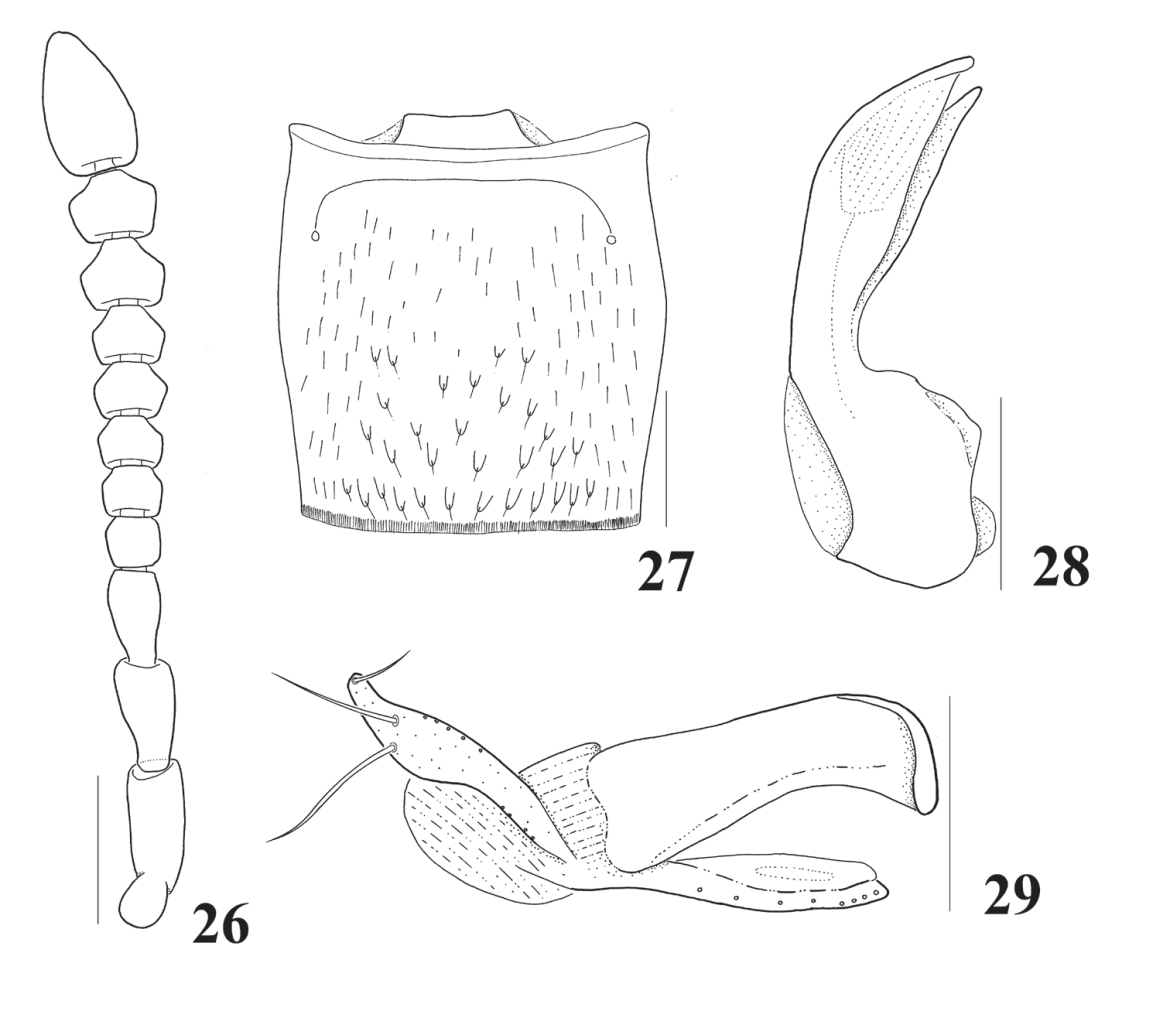
*Homalota sauteri*: **26** antenna **27** male tergite VII, dorsal aspect **28** median lobe, lateral aspect **29** paramere, lateral aspect. Scale bars 0.1 mm.

Body (Fig. 4) length about 1.4–1.7 mm. Body dorsoventrally strongly flattened, parallel-sided; surface glossy, pubescent; light brown, head and abdominal tergite VI brown. *Head.* Subquadrate, narrower than pronotum, eyes moderate in size, slightly shorter than tempora; antennomere 4 subquadrate, 5–10 transverse, incrassate toward (Fig. 26). *Mouthparts.* Labrum transverse, 7 pairs of macrosetae present, sensilla of antero-medial sensory area distinct, shallow and moderately emarginated, α-sensillum with a minute setose process, β and γ minute and conical, ε with a setose process, slightly longer than α, insertion more or less distant from anterior margin of labrum, two lateral sensilla present on lateral margins of epipharynx, without transverse row of sensory pores on basal region of epipharynx; right mandible with small median tooth, prostheca well developed, divided into 2 distinct area; maxillary palpomere 2 dilated distally, 3 distinctly dilated to apical third and then slightly convergent toward apex, 4 without small spine at apex; labium with ligula moderate in length and bifid at half, almost as long as labial palpomere 1, labial palpomere 1 longer than 2, two medial setae present on prementum, side by side and narrowly separated, median pseudopore field moderate and with pseudopores, mentum slightly emarginated in anterior margin. *Thorax.* Pronotum transverse, about 1.3 times wider than long, widest at basal third, surface pubescent, directed postero-laterally, without distinct macrosetae, hypomeron broadly visible in lateral aspect; prosternum with a distinct median knob; elytra wider than pronotum, postero-laterally sinuate; mesoventrite without longitudinal carina, mesoventral process narrow, apex pointed; metaventral process narrowly round at apex, shorter than mesoventral process; isthmus very slightly present, tarsomere 1 of front and middle legs as long as 2, 1 slightly longer than 2 in hind leg, with an empodial seta between tarsal claws. *Abdomen.* Tergites III–VI transversely impressed; tergite X with medial setal patch chevron shaped, with 4 macrosetae on each side. *Genitalia.* Spermatheca elongate at base, duct convoluted and coiled; median lobe (Fig. 28) elongate, bulbous at base, apical process elongate, distinctly longer than basal bulb, flagellum well sclerotized and short; paramere (Fig. 29) with apical lobe of paramerite long and subcylindrical, with four setae, 2 relatively longer than the others, condylite subequal in length to apex of paramerite. *Secondary sexual characteristics.* Postero-medial margin of male tergite VII (Fig. 27) with 16 to 18 tubercles; posterior margin of male tergite VIII emarginate at middle, female tergite VIII more or less round.

#### Distribution.

Korea (South), Japan, Taiwan.

#### Remarks.

*Homalota sauteri* is similar to *Homalota serrata*, but can be distinguished by the following features: body pubescent, head narrower than pronotum, male tergite VII with 16 to 18 tubercles. The taxonomic position of *Homalota sauteri* in *Homalota* is unclear and tentatively maintained here, pending further comprehensive research of this species.

### Homalota serrata

Taxon classificationAnimaliaColeopteraStaphylinidae

(Assing) comb. n.

Figs 5 , 30 – 36

Anomognathus serratus Assing, 2011: 306.

#### Specimens examined.

KOREA: Gangwon Prov., Sokcho-city, Mt. Seolak, Hwaamsa, 21 vi 2002, SJ Park, CW Shin, *ex* fungus on log (1♂ on slide); Gyeongnam Prov., Sacheon-si, Guam, 16 v 1986, KS Lee (2♀♀); Jeju Prov., Wimiri, 29 iii 1985, KS Lee, under bark (1♀); Tongyeong-si, Sanyang-eup, minam-ri, 34°46'20.69"N, 128°24'44.03"E 135, 15 IV 2011, YH Kim, under bark (1♂).

#### Description.

**Figures 30–36.**
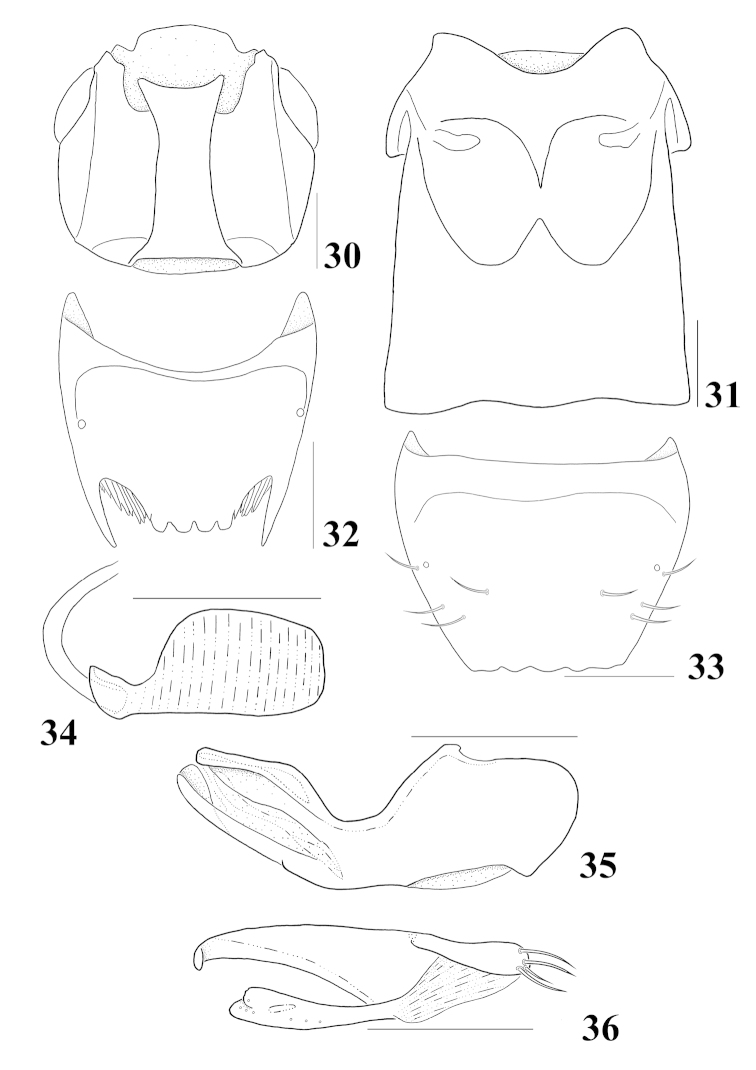
*Homalota serrata*: **30** head, ventral aspect **31** meso- and metaventrites, ventral aspect **32** male tergite VIII, dorsal aspect **33** female tergite VIII, dorsal aspect **34** spermatheca **35** median lobe, lateral aspect **36** paramere, lateral aspect. Scale bars 0.1 mm.

Body (Fig. 5) length about 1.4–2.0 mm. Body dorsoventrally strongly flattened, parallel-sided; surface glossy, slightly pubescent; yellowish brown, head and abdominal tergite VI dark brown. *Head.* (Fig. 30) Subquadrate; eyes moderate in size, almost as long as tempora; antennomeres 5–10 transverse; incrassate toward. *Mouthparts.* Labrum transverse, 7 pairs of macrosetae present; α-sensillum with a setose process, β and γ minute and conical, ε with a minute setose process, two lateral sensilla present on lateral margins of epipharynx, transverse row of sensory pores absent on basal region of epipharynx; right mandible with median tooth, prostheca well developed, divided into 3 distinct area; maxillary palpomere 2 and 3 dilated distally, 4 without small spines at apex; labium with ligula moderate in length and bifid at half, as long as labial palpomere 1, labial palpomere 1 longer than 2, two medial setae present on prementum, contiguous, side by side, median pseudopore field of prementum narrow and with pseudopores, mentum slightly emarginated in anterior margin. *Thorax.* Pronotum slightly transverse, about 1.2 times wider than long, widest at apical third, surface pubescent, directed posteriorly in narrow median strip and directed postero-laterally to laterally in lateral area; hypomeron broadly visible in lateral aspect; prosternum with a median knob; elytra slightly wider than pronotum, postero-laterally slightly sinuate; mesoventrite (Fig. 31) without longitudinal carina, mesoventral process narrow, apex acute; metaventral process round at apex, almost as long as mesoventral process; isthmus present; mesocoxae narrowly separated; tarsomere 1 as long as 2 in front leg, 1 longer than 2 in middle and hind legs, without empodial seta between tarsal claws. *Abdomen.* Tergites III–V transversely impressed; medial setal patch of tergite X with medial setal patch subquadrate, with transverse row of setae anteriorly. *Genitalia.* Spermatheca (Fig. 34) simple and round at base; median lobe (Fig. 35) elongate, bulbous at base, apical process slender and short, distinctly shorter than basal bulb, flagellum well sclerotized and short; paramere (Fig. 36) with apical lobe of paramerite subcylindrical, with four setae, 2 setae longer than others, condylite shorter than in length to apex of paramerite. *Secondary sexual characteristics.* Posterior margin of male tergite VIII (Fig. 32) with two long lateral processes, apex acute, median area with 4 short and broad processes, several long spines placed between them, posterior margin of female tergite VIII (Fig. 33) truncate and serrate.

#### Distribution.

Korea (South), China (Zhejiang).

#### Remarks.

*Homalota serrata* can be distinguished from other Palearctic *Homalota* species by the following features: body slightly pubescent, head as wide as pronotum, male tergite VII without tubercles and distinct structure of tergite VIII in both sexes.

Assing (2011) described *Anomognathus serratus* from China (Zhejiang Province). However, we propose that *Anomognathus serratus* be placed in the genus *Homalota*, based on the following characters: infraorbital carinae complete; medial setae of prementum contiguous; scutellum posteromedially round; mesocoxae narrowly separated; isthmus distinctly less than half of the mesocoxae length. According to article 34.2 of the International Code of Zoological Nomenclature (ICZN), the name *serratus* must agree in gender with the generic name *Homalota* Mannerheim (feminine); therefore, it is corrected to *serrata*.

## Supplementary Material

XML Treatment for Homalota

XML Treatment for Homalota fraterna

XML Treatment for Homalota mikado

XML Treatment for Homalota plana

XML Treatment for Homalota sauteri

XML Treatment for Homalota serrata

## References

[B1] Ashe JS (1984) Generic revision of the subtribe Gyrophaenina (Coleoptera: Staphylinidae: Aleocharinae) with a review of the described subgenera and major features of evolution. Quaestiones Entomologicae 20: 129–349

[B2] Assing V (2011) Six new species and additional records of Aleocharinae from China (Coleoptera: Staphylinidae: Aleocharinae). Linzer Biologische Beitraege 43(1): 291–310

[B3] Bernhauer M (1907) Zur Staphylinidenfauna von Japan. Verhandlungen der Kaiserlich-Königlichen Zoologisch–Botanischen Gesellschaft in Wien 57: 371–414

[B4] Fenyes A (1914) H. Sauter‘s Formosa–Ausbeute. Aleocharinae. Archiv für Naturgeschichte (A) 80: 45–55

[B5] Fenyes A (1918) Coleoptera Fam. Staphylinidae subfam. Aleocharinae. In: Wytsman P (Ed.) Genera Insectorum. Fascicle 173a. Louis Desmet-Verteneuil, Bruxelles, 110 pp

[B6] Gyllenhal L (1810) Insecta Suecica descripta. Classis I. Coleoptera sive Eleuterata. Tomi I. Pars II. L. J. Leverentz, Scaris, 660 pp

[B7] Likovský Z (1984) Über die Nomenklatur der Aleocharinen (Coleoptera, Staphylinidae). Annotationes Zoologicae et Botanicae 160: 1–8

[B8] Mannerheim CG (1830) Précis d'un nouvel arrangement de la famille des brachélytres, de l'ordre des insectes coléoptères. St. Petersbourg, 87 pp

[B9] Newton AF Thayer MK (2005) Catalog of higher taxa, genera, and subgenera of Staphyliniformia [online]. Field Museum of Natural History, Chicago. http://www.fieldmuseum.org/peet_staph/db_1d.html [last updated November 3, 2005; accessed/downloaded May 29, 2012]

[B10] Paśnik G (2001) The North Korean Aleocharinae (Coleoptera, Staphylinidae): diversity and biogeography. Acta Zoologica Cracoviensia 44: 185–234

[B11] Sawada K (1972) Methodological Research in the Taxonomy of Aleocharinae. Contributions from the Biological Laboratory Kyoto University 24(1): 31–59

[B12] Sharp DS (1888) The Staphylinidae of Japan. The Annals and Magazine of Natural History 2(6): 227–295, 369–387, 451–464

[B13] Smetana A (2004) Family Staphylinidae: Aleocharinae. In: Löbl I Smetana A (Eds) Catalogue of Palaearctic Coleoptera Volume 2 Hydrophiloidea-Histeroidea-Staphylinoidea. Apollo Books, Stenstrup, 353–494

